# Supplementary material for: Recycling of memory B cells between germinal center and lymph node subcapsular sinus supports affinity maturation to antigenic drift
Source: Nat Commun. 2022 May 5;13:2460. doi: 10.1038/s41467-022-29978-y (PMC9072412; doi:10.1038/s41467-022-29978-y)
Supplement: Supplementary file 1 — Supplementary Information [file 41467_2022_29978_MOESM1_ESM.pdf]

Supplementary Information

Recycling of memory B cell between germinal center and lymph node subcapsular sinus supports affinity maturation to antigenic drift

Yang Zhang[1, 13], Laura Garcia-Ibanez[1, 13], Carolin Ulbricht[2], Laurence S C Lok[3], Jeremy A Pike[4], Jennifer Mueller-Winkler[5], Thomas W Dennison[3], John R Ferdinand[3], Cameron J M Burnett[1], Juan C Yam-Puc[1], Lingling Zhang[1, 5], Raul Maqueda Alfaro[1, 6], Yousuke Takahama[7], Izumi Ohigashi[8], Geoffrey Brown[6], Tomohiro Kurosaki[9, 10], Victor L J Tybulewicz[5, 11], Antal Rot[12], Anja E Hauser[2], Menna R Clatworthy[3], Kai-Michael Toellner[1]

[1] Institute of Immunology and Immunotherapy, College of Medical and Dental Sciences, University of Birmingham, Birmingham, UK. [2] Department of Rheumatology and Clinical Immunology, Charité - Universitätsmedizin Berlin, corporate member of Freie Universität Berlin and Humboldt-Universität zu Berlin, 10117 Berlin, Germany, and Deutsches Rheuma-Forschungszentrum (DRFZ), a Leibniz Institute, Charitéplatz 1, 10117 Berlin, Germany, [3] University of Cambridge Molecular Immunity Unit, MRC Laboratory of Molecular Biology, Cambridge Biomedical Campus, Cambridge, UK, [4] Centre of Membrane Proteins and Receptors (COMPARE), Universities of Birmingham and Nottingham, UK, and Institute of Cardiovascular Sciences, College of Medical and Dental Sciences, University of Birmingham, UK [5] The Francis Crick Institute, London, UK, [6] Department of Cell Biology, Center for Research and Advanced Studies, The National Polytechnic Institute, Cinvestav-IPN, Av. IPN 2508, San Pedro Zacatenco, Gustavo A. Madero, 07360 Mexico City, Mexico, [7] Thymus Biology Section, Experimental Immunology Branch, National Cancer Institute, National Institutes of Health, Bethesda, MD, 20892, USA, [8] Division of Experimental Immunology, Institute of Advanced Medical Sciences, University of Tokushima, Tokushima, 770-8503, Japan, [9] Laboratory of Lymphocyte Differentiation, WPI Immunology Frontier Research Center, Osaka University, Osaka 565-0871, Japan, [10] Laboratory of Lymphocyte Differentiation, RIKEN Center for Integrative Medical Sciences (IMS), Yokohama, Kanagawa 230-0045, Japan, [11]Imperial College, London W12 0NN, UK, [12]Centre for Microvascular Research, The William Harvey Research Institute, Queen Mary University London, EC1M 6BQ London, UK; Centre for Inflammation and Therapeutic Innovation, Queen Mary University London, EC1M 6BQ London, UK; Institute for Cardiovascular Prevention, Ludwig-Maximilians University, 80336 Munich, Germany.[13] Contributed equally.

Correspondence to:
Kai-Michael Toellner, PhD
Institute of Immunology and Immunotherapy
College of Medical and Dental Sciences
The University of Birmingham
B15 2TT UK
e-mail: k.m.toellner@bham.ac.uk
phone: +44 121 415 8681
Fax: +44 121 41 43599

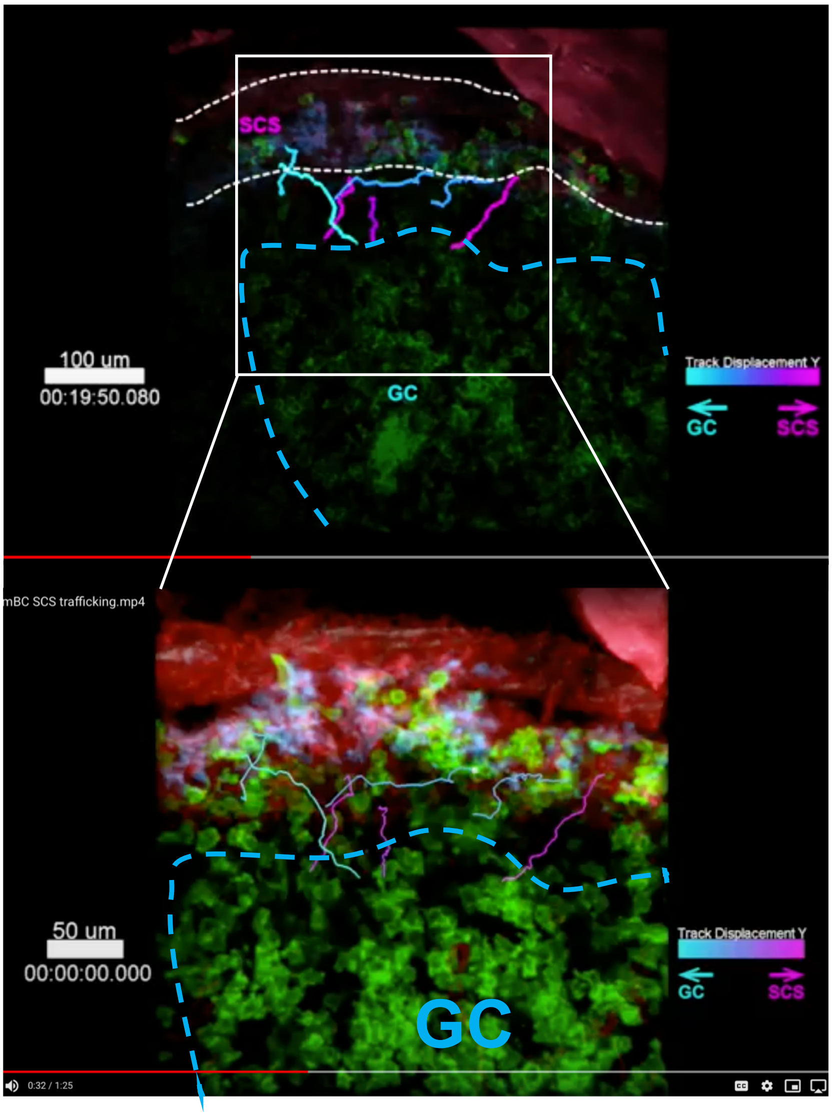

Supplementary Figure 1: B_{EM} recycling between GC and SCS

Still image from intravital microscopy videos of Cγ1Cre mTmG ACKR4$^{+/+}$ in drLN 8 d after foot immunization. Prior recombination, Cγ1Cre mTmG mice express membrane targeted tdTomato (mT) in all body cells. Following B cell activation, Cre expression through induction of IgG1 heavy chain germline transcripts leads to excision of the loxp-flanked mT gene and expression of membrane targeted eGFP (mG)[1]. mG-labelled B_{EM} (green), mT-labelled stroma (red) and CD169 labelled SCS macrophages (blue). B_{EM} were manually tracked moving from the GC towards the SCS (pink tracks) or recycling from the SCS to the GC (blue tracks). Representative image of 7 Cγ1Cre mTmG ACKR4$^{+/+}$ mice.

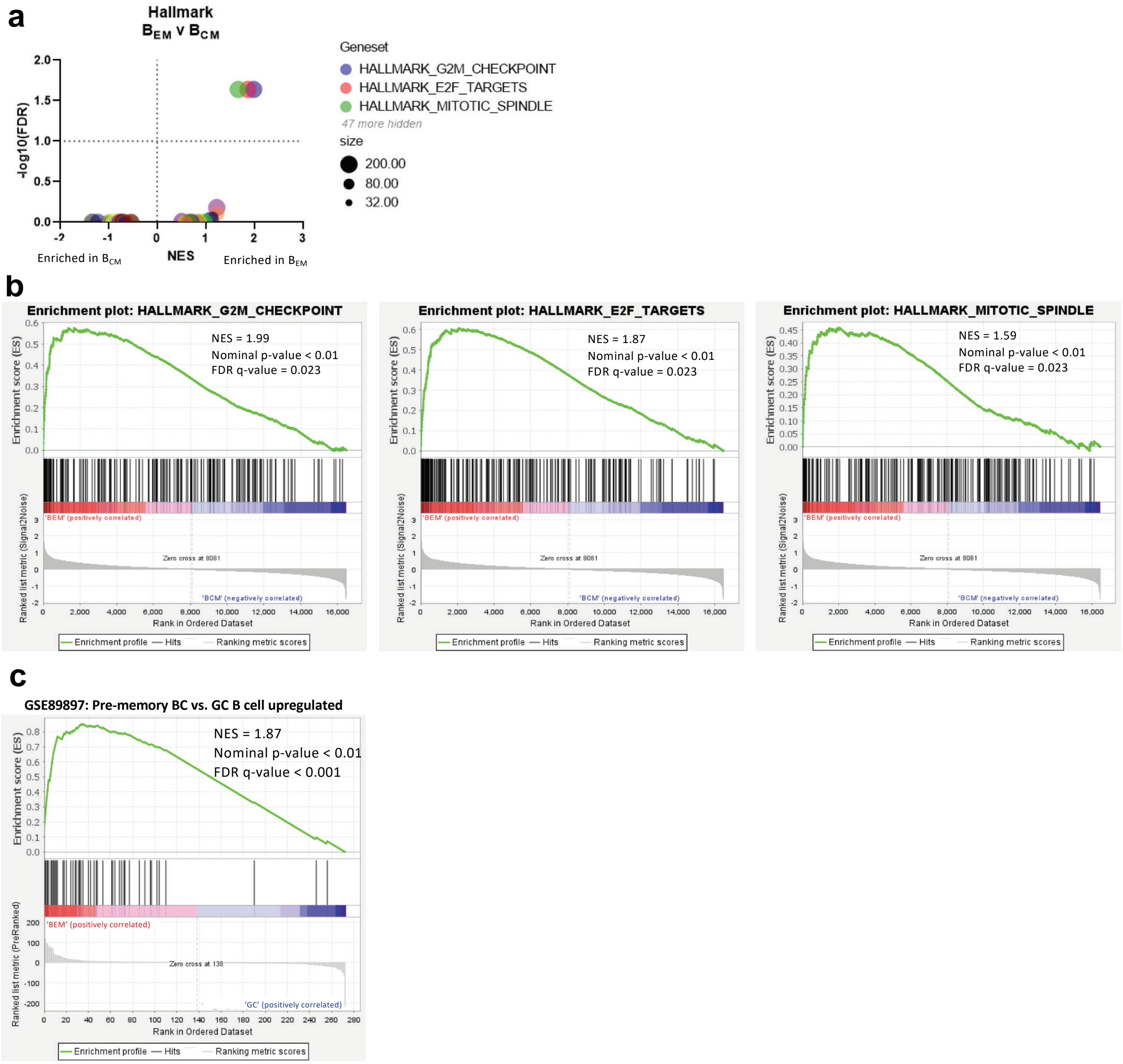

Supplementary Figure 2: Gene set enrichment analysis (GSEA)
RNA sequencing data was aligned to GRCm38 genome and counted by STAR 2.7.3a. Gene set enrichment analysis was performed on emigrating memory B cells (B_{EM}) compared with circulating memory B cells (B_{CM}) using the Hallmark gene set collection [2]. GSEA software used was GSEA 4.1.0 (Broad Institute). **a** Bubble plot represents complete Hallmark GSEA of BEM v BCM normalized counts. Each point is scaled to the size of the gene set (after being restricted to the data set). NES: Normalized enrichment score. Figure legend lists significantly enriched gene sets (FDR < 0.1). **b** Gene set enrichment plots of significantly enriched gene sets from Hallmark GSEA of BEM v BCM. **c** RNA sequencing data was counted and aligned to GRCm38 genome by Partek Flow. Pre-ranked dataset isolated from gene specific analysis of BEM v GC with P-value < 0.05 and ranked by fold change. GSEA was performed using this pre-ranked list against the pre-memory B cell gene expression profile from Laidlaw et al.[3] using genes upregulated in pre-memory B cells compared GC B cells [3]. Raw counts data accessed from gene expression omnibus from GSE89897, with pre-memory B cells defined as Ephrin-B1^{+} S1PR2-Venus^{-} B cells in drLN and GC B cells defined as Ephrin-B1^{+} S1PR2-Venus^{+} B cells in drLN.

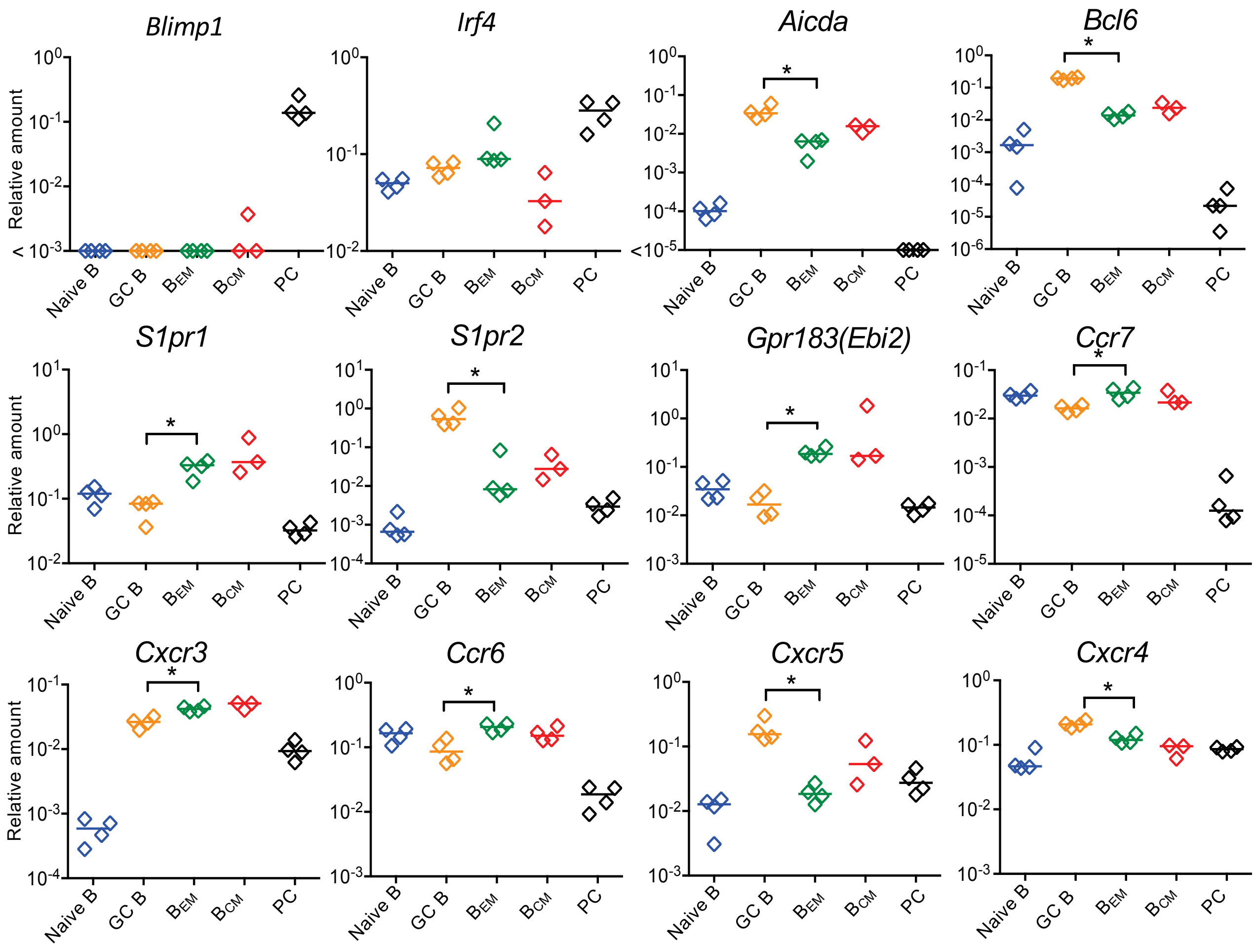

Supplementary Figure 3: Gene expression on different B cell populations.

Selected genes expressed on different populations FACS sorted from popliteal lymph node in C57BL/6J mice that had received NP+ B cells from Cγ1Cre QM mT/mG, 8 d after foot injection.

Each diamond represents pooled cells from both popliteal drLN nodes of 4 mice, representative of 3 independent experiments (total n=12 mice). All values are relative to *b2m* mRNA. Two-tailed Mann-Whitney testing. Statistical significance (*: $p=0.0286$) is only indicated for gene expression differences between GC BC and B_{EM}.

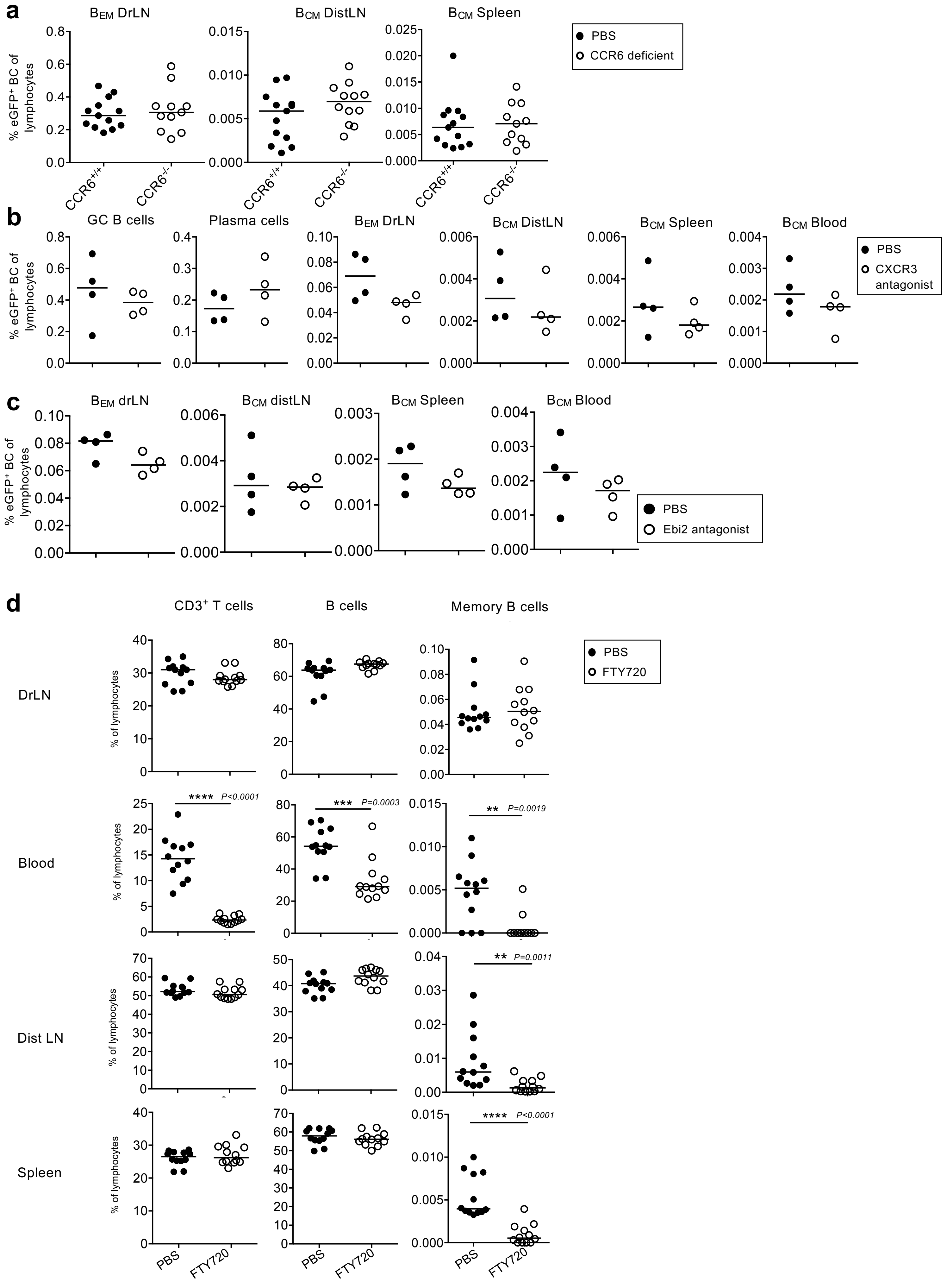

Supplementary Figure 4: Effect of blocking S1PR, CCR6, CXCR3, or EBI2 signals.

Supplementary Figure 4: Effect of blocking CCR6, CXCR3, EBI2, or S1PR signals.

a Effect of CCR6 deficiency for the production of B_{EM}. NP-specific B cells from Cγ1Cre QM mTmG or Cγ1Cre QM mTmG CCR6 ko mice were transferred into C57BL/6J recipients separately, followed by foot immunization with NP-CGG in alum. Gating scheme for $eGFP^+$ B emerging from GC (B_{EM}) in draining LN (drLN) and $eGFP^+$ circulating B cells (B_{CM}) in distant LN (distLN) and spleen were measured by flow cytometry at 8 d after immunization. Data pooled from 3 independent experiments with 3-4 mice each (total n=12). **b** Effect of CXCR3 blockade on the production of B_{EM}. NP-specific B cells from Cγ1Cre QM mTmG mice were transferred into C57BL/6J recipients, followed by foot immunization with NP-CGG in alum one day later. $eGFP^+$ B_{EM} in draining LN (drLN) and $eGFP^+$ B_{CM} in distLN, spleen, and blood were measured by flow cytometry at 5 h after NBI74330 (CXCR3 antagonist, 60 ug / mouse) treatment via i.p. at 8 d after NP-CGG immunization. Each symbol represents one mouse. Data from one experiment (n=4). **c** Effect of Ebi2 blockade for the production of memory B cells. NP-specific B cells from Cγ1Cre QM mT/mG mouse were transferred into C57BL/6J recipients separately, followed by foot immunization with NP-CGG in alum. $eGFP^+$ B emerging from GC (B_{EM}) in draining LN (drLN) and $eGFP^+$ circulating B cells (B_{CM}) in distant LN (distLN) and spleen were measured by flow cytometry at 5hr after NIBR189 (Ebi2 antagonist, 60ug/mouse) treatment via i.p. at 8 d after NP-CGG immunization. Each symbol represents one mouse. Data from one experiment (n=4). **d** Effect of S1PR on the migration of naïve T helper and B cells, and memory B cells. Mice received FTY720 over 2 d before tissue collection. Total $CD3^+$ T cells, $B220^+$ B cells, and B_{EM} in draining lymph node or B_{CM} in blood, distant lymph nodes, and spleens were analyzed by flow cytometry. Each symbol represents one mouse. Data merged from three independent experiments (total n=12). Two-tailed Mann-Whitney test.

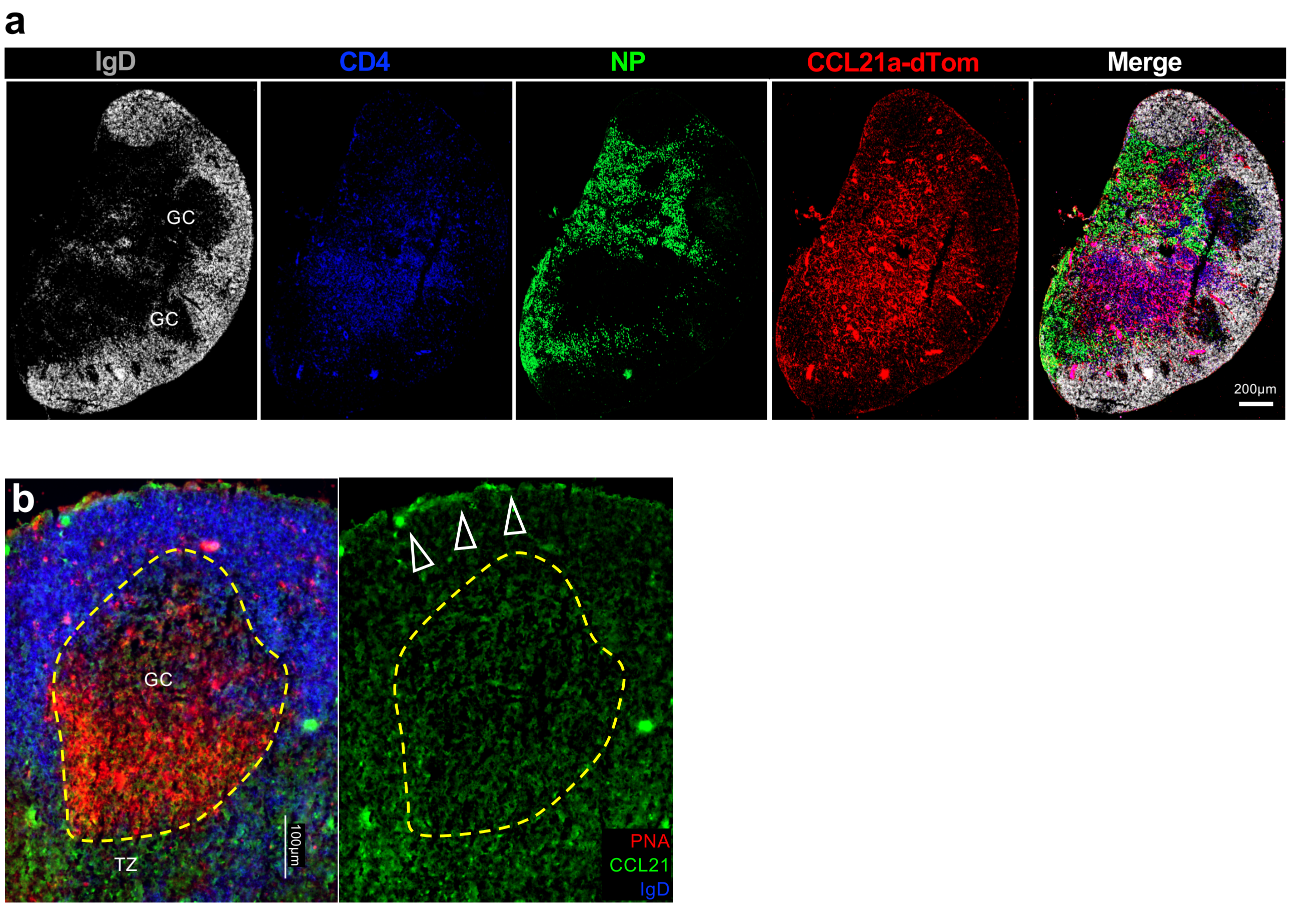

Supplementary Figure 5: CCL21 expression in and under the SCS floor endothelium.

a Popliteal lymph node from a heterozygous *Ccl21*^{tdTom} reporter mouse 8 d after foot immunization with NP-CGG showing CCL21a expression in SCS floor endothelium and stromal cells in the follicle. tdTomato (red), immunohistology for IgD to indicate follicles and GCs (grey), CD4 for T zone (blue), NP-specific binding on plasma cells and GC (green). Arrowheads: Ccl21 expression close to GCs.

Image is a representative from 4 lymph nodes. **b** CCL21 protein immunohistology of drLN 8 d after foot immunization showing stronger CCL21 signal close to SCS (arrowheads) and in the T zone. Representative image from 4 ACKR4$^{+/+}$lymph nodes

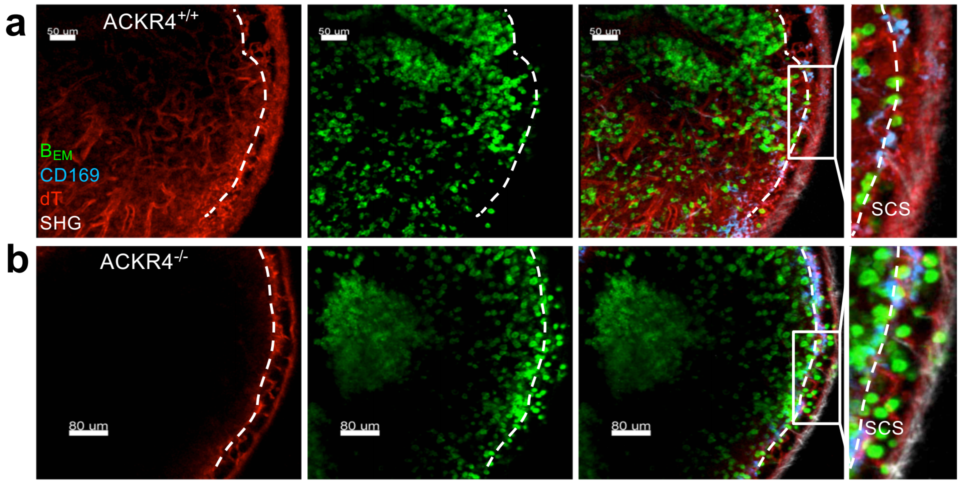

Supplementary Figure 6: More B_{EM} in SCS of ACKR4 deficient drLN

Still images of intravital microscopy of Cγ1Cre mTmG ACKR4$^{+/+}$ (**a**) and ACKR4$^{-/-}$ (**b**) in drLN 8 d after foot immunization. This shows eGFP-labelled B_{EM} (green), mTomato-labelled stroma (red) and CD169 labelled SCS macrophages (blue). 10 µm-thick sections are shown, with stromal signal strength dependent on tissue depth. Note larger numbers of B_{EM} in the SCS in ACKR4$^{-/-}$ environment. Representative images of 7 ACKR4$^{+/+}$ and 3 ACKR4$^{-/-}$ mice.

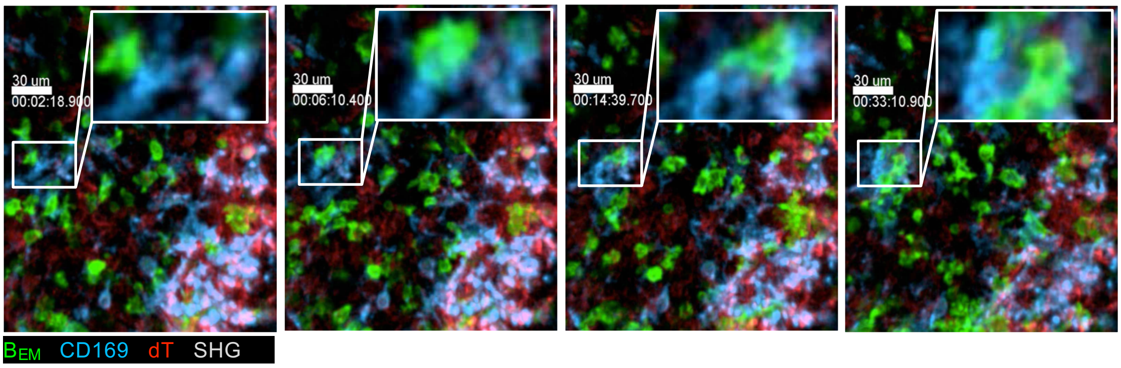

Supplementary Figure 7: Intravital observation of B_{EM} interacting with CD169⁺ SCS macrophage

Still images from intravital microscopy of SCS in Cγ1Cre mT/mG ACKR4$^{+/+}$ drLN 8 d after foot immunization. eGFP^{+} B_{EM} make prolonged interaction with CD169-labelled SCS macrophages (blue). mTomato positive stroma (red). Representative images of 7 in Cγ1Cre mT/mG ACKR4$^{+/+}$ mice.

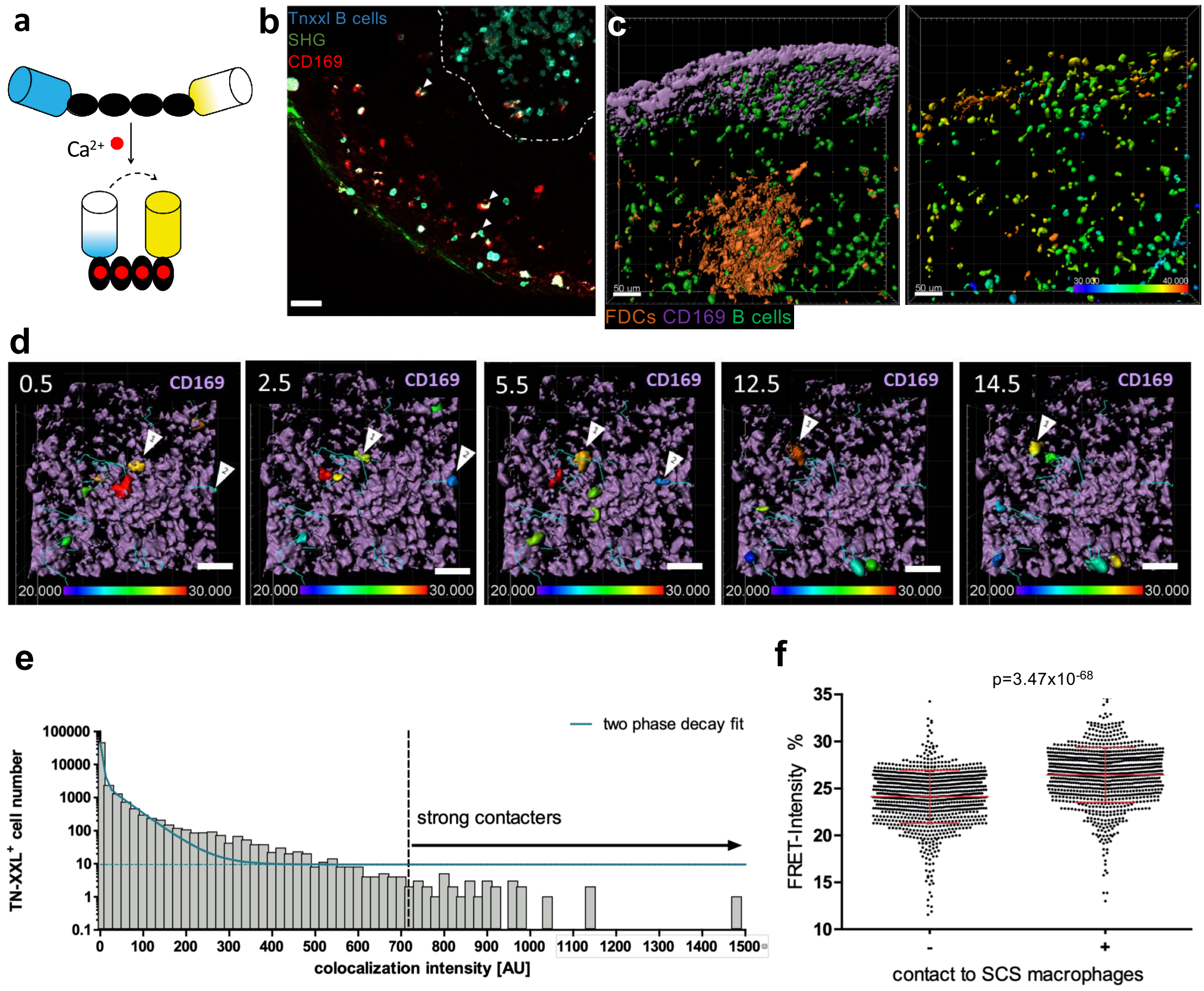

Supplementary Figure 8: Intravital observation of intracellular Ca^{2+} in B_{EM}

a Schematic representation of the genetically encoded calcium indicator TN-XXL. The four troponin c binding sites of TN-XXL (black ovals) can be loaded with four calcium ions (red) that cause a conformational change. Förster resonance energy transfer leads to decreased eCFP (blue) and increased citrine (yellow) fluorescence after multiphoton excitation with 850nm laser light. **b** Single z plane of intravitally imaged lymph node with capsule visible (SHG), CD169-efluor660 stained macrophages (red) and adoptively transferred TN-XXL+ B1-8^{hi} (NP-specific) B cells (cyan). A germinal center is visible at the upper right (broken white line). Contacts between CD169+ macrophages and TN-XXL^{+} B cells are shown in white (white arrows). **c** Left: Surface rendering of subcapsular sinus (light purple, CD169-stained macrophages) and germinal center (light orange, FDC staining with CD21/35-Atto590), TN-XXL^{+} B1-8^{hi} B cells (green). Right: FRET intensity of TN-XXL^{+} B cells shown in left image, color-coded for mean FRET intensity. Scale bar 50 µm. **d** Stills of TN-XXL^{+} cells tracked over 30 minutes, mean FRET values depicted as color coding. $CD169^{+}$ macrophages in light purple, track lines in cyan. White arrows point to tracks analyzed in (**e**) and (**f**) for FRET and colocalization intensity over time. Scale bar 40 µm. **e** Colocalization histogram and two-phase decay fit for analysis of colocalization between $CD169^{+}$ macrophages and TN-XXL^{+} B cells. The colocalization intensity where the decay reaches a plateau was determined as threshold for strong (+) macrophage-to-B-cell contact. B cells with colocalization intensity of 0 AU were assigned to the (-) group. **f** Plot of a random selection of 1,000 mean FRET intensities (total 63,000 events) of surface rendered B cells gated on contact strengths to SCS macrophages: (-) group: colocalization intensity = 0 AU; (+) group: colocalization intensity > 717 AU. One dot per TN-XXL+ object. Plot contains data from 5 animals from two different experiments. Student's two-tailed t-test with Welch correction. Data show mean and standard deviation.

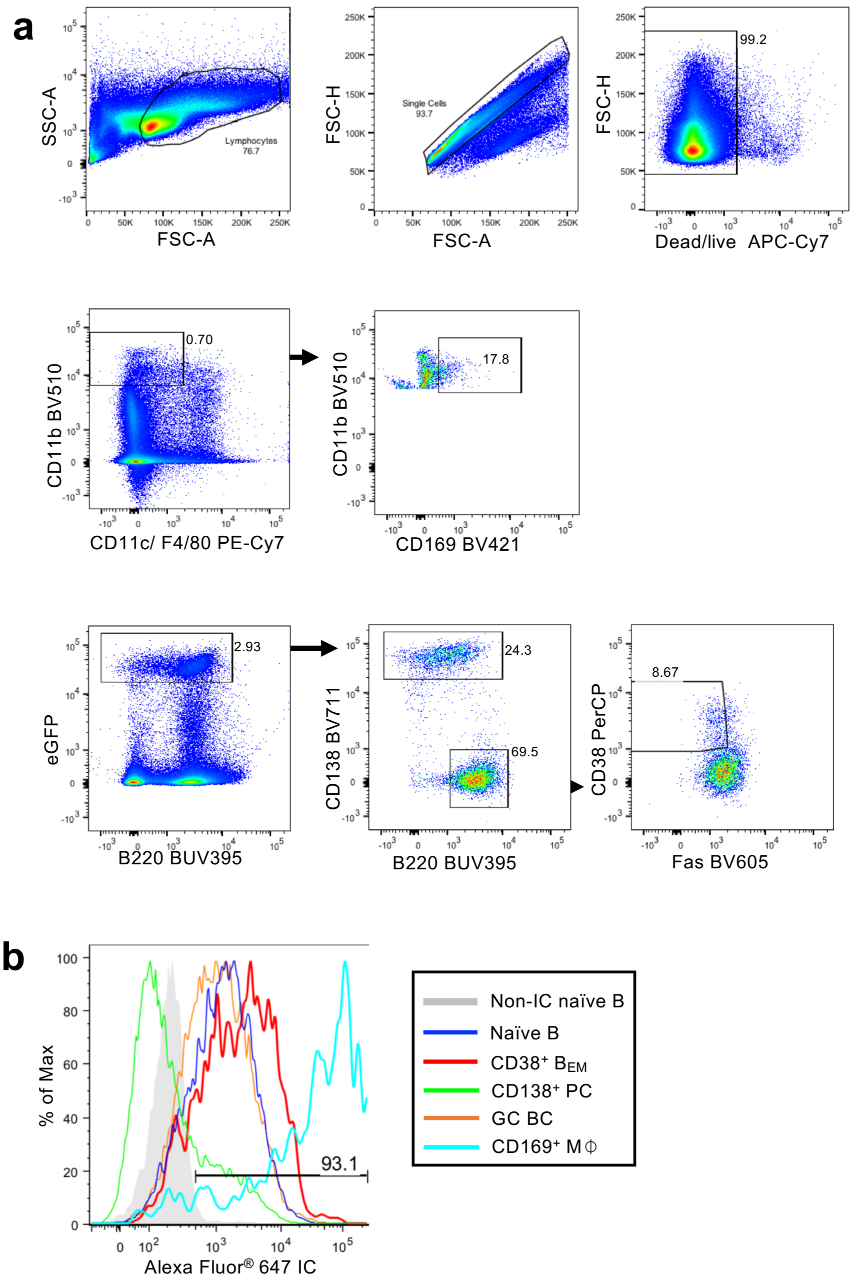

Supplementary Figure 9: Gating scheme for F4/80$^-$CD11b$^+$ CD169$^+$ SCS macrophages

a The gating scheme for the flow cytometric detection of F4/80$^-$CD11b$^+$CD169$^+$ SCS macrophages[4], and eGFP$^+$ $B_{EM,}$ GC B cell, and plasma cells from drLN is shown. Cγ1Cre mTmG mice 10 min after foot injection with Alexa647 labelled immune complex (IC) at 8 d after foot immunization. **b** FACS histogram showing Alexa647 IC staining on different

.

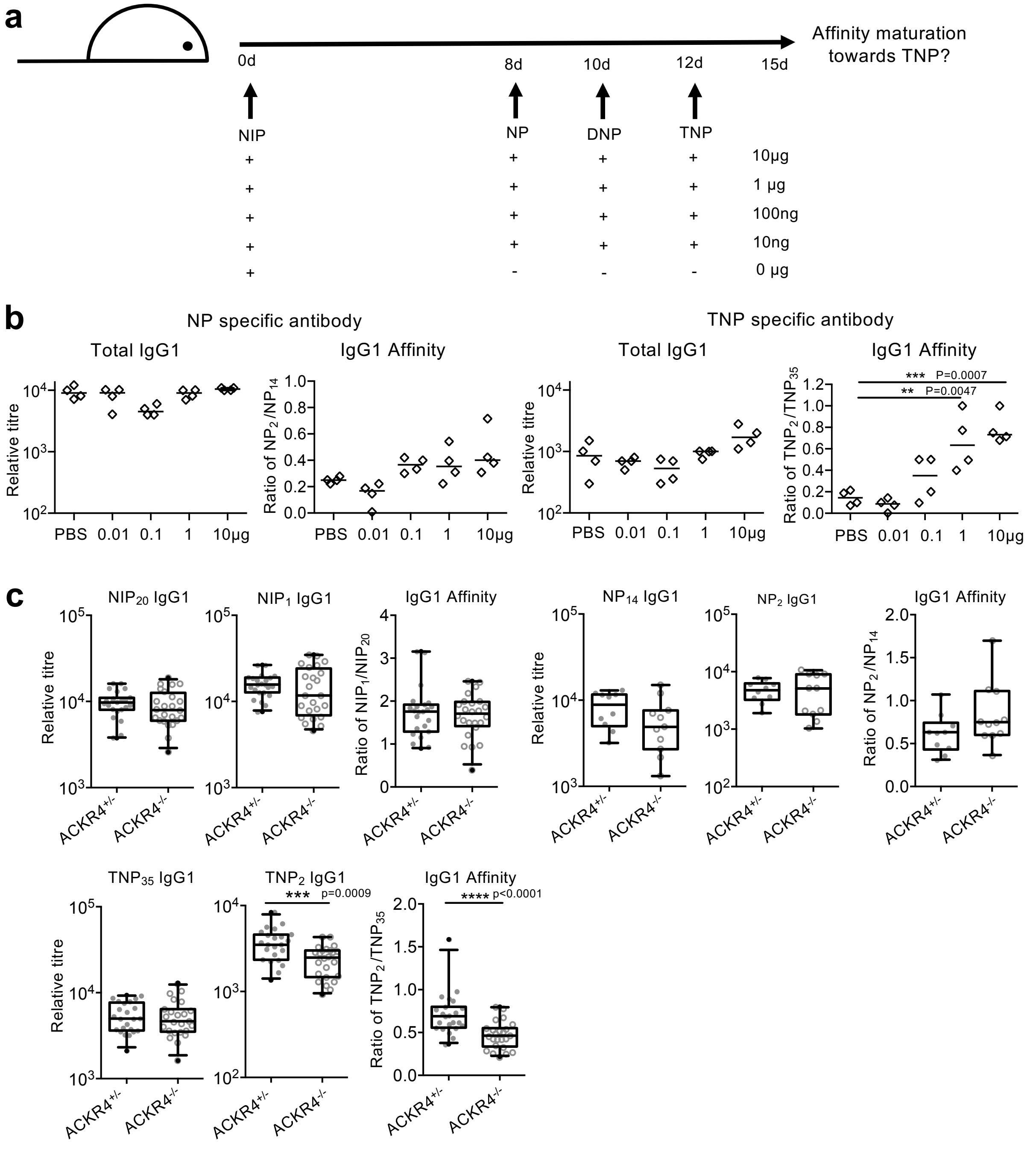

Supplementary Figure 10: Antigenic drift experiment

a Experimental design to simulate antigenic drift. Wild type mice were primed with 20 µg / 20 µl NIP-KLH in alum with B. pertussis into foot, followed by subsequent injections 8, 10, and 12 d after immunization with soluble NP-, DNP-, TNP-KLH into the same foot respectively. Control mice received mock immunization. Blood was collected 15 days post-immunization. **b** NP- or TNP-specific antibody titer and affinity. Each dot represents one animal. ANOVA with Tukey test for multiple comparisons, ** p=0.0047, *** p=0.0007. **c** NIP-, NP-, or TNP-specific antibody titers and affinity after immunization of $ACKR4^{+/-}$ or $ACKR4^{-/-}$ litter mates with NIP-KLH followed by NP-, DNP-, TNP-KLH. Merged data from 4 independent experiments with 5 mice each (total n=20). Box plots show medians, boxes: 50%, whiskers: 5-95 %. Symbols represent individual mice. Two-tailed Mann-Whitney test, *** p=0.0009, **** p<0.0001.

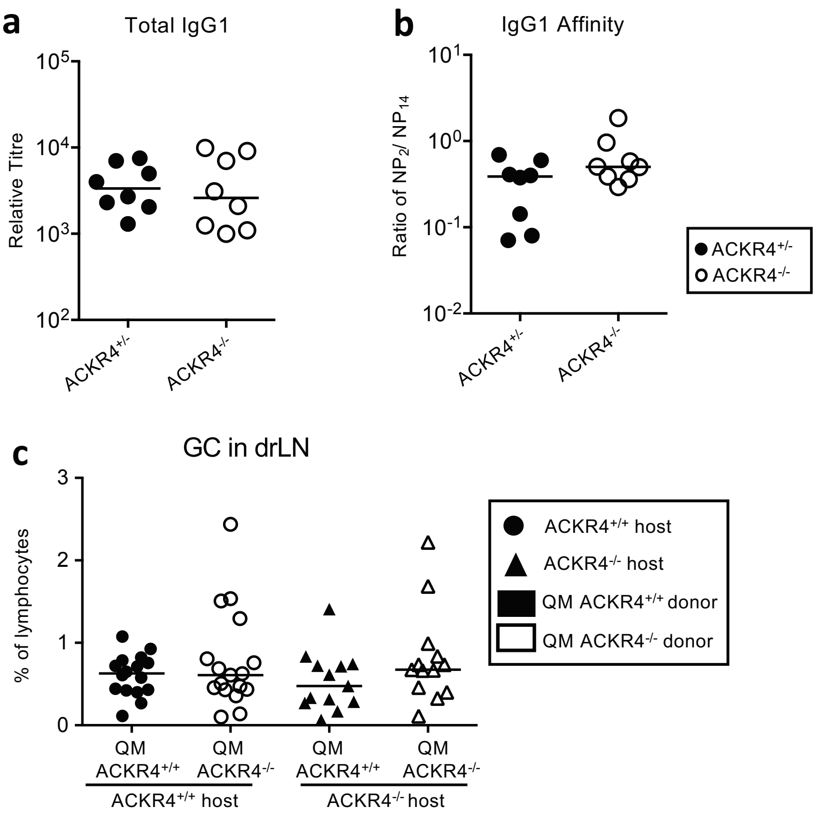

Supplementary Figure 11: No major changes in antibody titers or antibody affinity in ACKR4-/- mice after NP-CGG immunization

a Relative NP-specific IgG1 antibody levels in serum of ACKR4+/- and ACKR4-/- mice 14 d post NP-CGG foot immunization. **b** Affinity of NP-specific IgG1. Plots from 2 independent experiments with 3-6 mice. Data from 14 d post primary immunization were combined from 2 independent experiments with 4 mice per group (total n=8). Each symbol represents one mouse. Horizontal line represents the median. **c** $ACKR4^{+/+}$ or $ACKR4^{-/-}$ hosts were adoptively transferred with a mix of the same numbers of NP-specific B cells from QM $ACKR4^{+/+}$ ($mTomato^{+}$) and from QM $ACKR4^{-/-}$ ($eYFP^{+}$) *i.v.*. Chimeras were foot immunized 24 h later with NP-CGG in alum. Sizes of GC was tested 8 days post-immunization in the drLN by flow cytometry, gating on $B220^{+}$ $CD38^{low}$ FAS^{+} and $eYFP^{+}$ or $mTomato^{+}$ GC B cells.

The data are merged from 3 independent experiments with 4-5 mice in each experiment (total n=13-16).

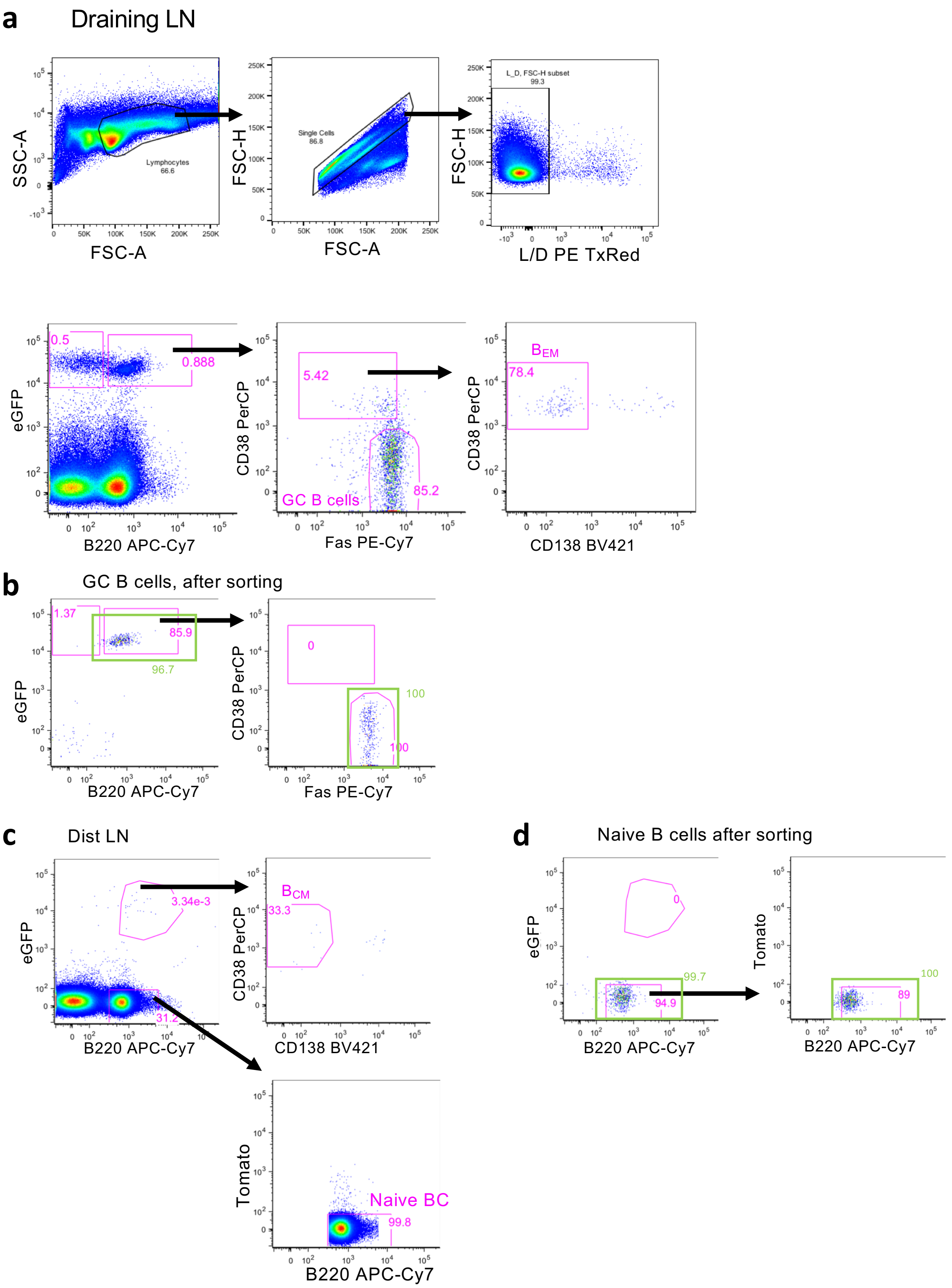

Supplementary Figure 12: Gating scheme for qRT-PCR, RNA-seq library preparation

FACS sorted cells from popliteal lymph node in C57BL/6J mice that had received NP$^+$ B cells from Cγ1Cre QM mT/mG, 8 d after foot injection. **a** eGFP$^+$B220$^+$Fas$^+$CD38$^-$ as GC B cells, and eGFP$^+$ B220$^+$ CD38$^+$Fas^{int} CD138$^-$ as B_{EM} cells in drLN. **b** GC B cell reanalysis was run on an independent FACS sorter. **c** eGFP$^+$ B220$^+$ CD38$^+$ Fas$^-$ CD138$^-$ as B_{CM}, and eGFP$^-$ Tomato$^-$ B220$^+$ as naive B cells in distLN. **d** Naive B cells after the sorting was run on an independent FACS sorter. Pink boxes and numbers: sorting gates and population size in %. Green boxes: reanalysis gates with purities in % of parent population.

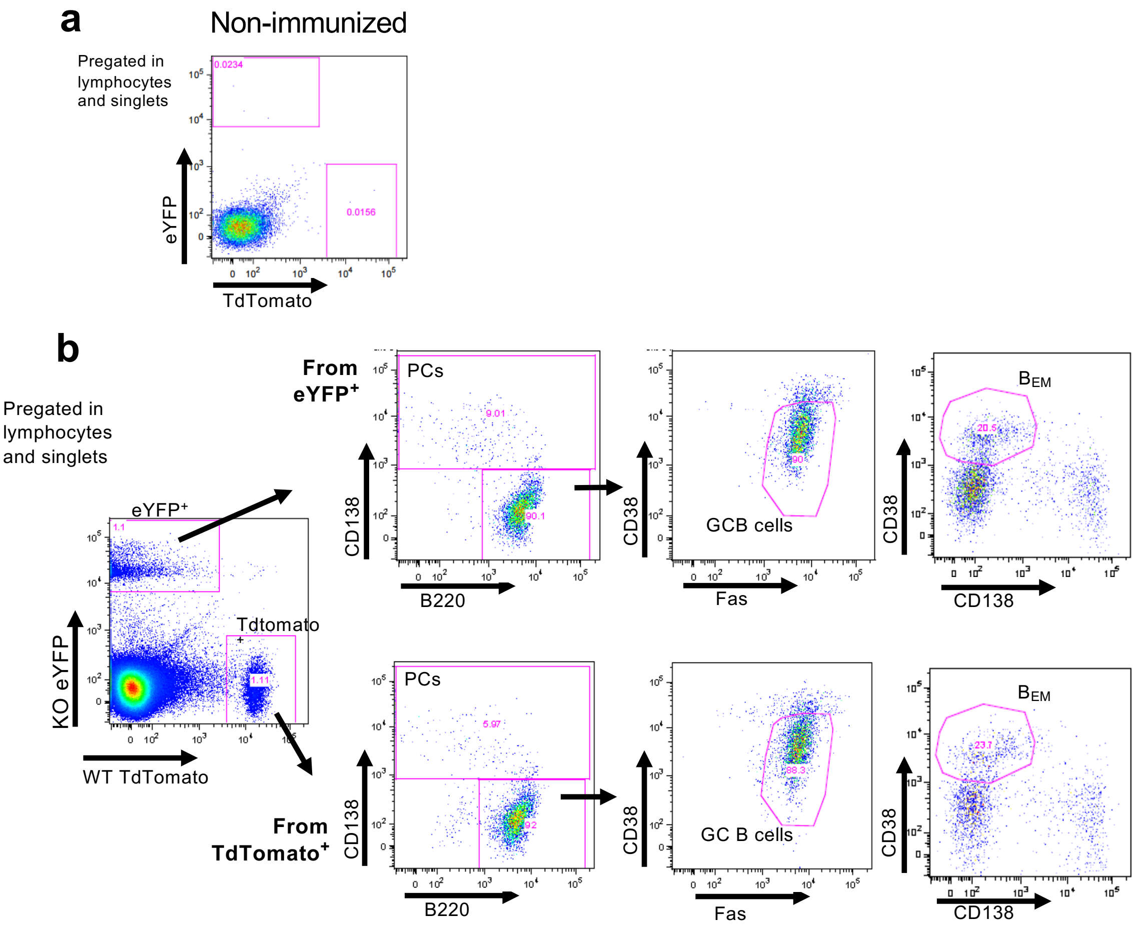

Supplementary Figure 13: Gating scheme for GC B cells and plasma cells, B_{EM} after co-transfer of B cells from QM ACKR4$^{+/+}$ and QM ACKR4$^{-/-}$ into ACKR4$^{+/+}$ or ACKR4$^{-/-}$ hosts.

1x10^5 NP$^+$B220$^+$ cells from QM TdTomato ACKR4$^{+/+}$ and 1x10^5 NP$^+$B220$^+$ cells from QM eYFP ACKR4$^{-/-}$ were co-transferred to ACKR4$^{+/+}$ or ACKR4$^{-/-}$ hosts *i.v.* and immunized 24 h later with NP-CGG into the rear feet. The response was studied 8 days post-immunisation.
a Representative example of a non-immunized host 8 days post-transfer with 1x10^5 NP$^+$B220$^+$ cells from each genotype.
b Representative diagram for flow cytometry gating of plasma cells, GC B cells, DZ GC B cells and LZ GC B cells, B_{EM} in the draining lymph node.
KO: QM eYFP ACKR4$^{-/-}$; WT: QM TdTomato ACKR4$^{+/+}$; PCs: plasma cells; GC: germinal center

Supplementary Table1: Primary Antibody list

Specificty	Clone	Source	Reference	RRID	Conjugation	Dilution
CD4	RM4-5	eBioScience	17-0042-82	AB_469323	APC	1/100 IHC
CD138	281-2	BD Biosiences	553712	AB_394998	purified	1/100 IHC
CD31	MEC13.3	BioLegend	102504	AB_312911	Biotin	1/200 IHC
IgD	Polyclonal	Novus Biologicals	NBP2-69334		purified	1/1000 IHC
IgD	11-26c.2a	BD Biosiences	553438	AB_394858	Purified	1/1000 IHC
IgD	11-26c.2a	BD Biosiences	560868	AB_10612002	APC	1/100 IHC, 1/1000 FACS
IgD	11-26c.2a	BD Biosiences	744291	AB_2742121	BV421	1/100 IHC, 1/200 FACS
Fibroblasts monoclonal antibody	ER-TR7	Invitrogen	MA1-40076		purified	1 in 50 IHC
Peanut Agglutinini (PNA)		Vector	B-1075-5		Biotin	1/200 IHC
LYVE1	Polyclonal	Abcam	ab14919		purified	1/200 IHC
Ki67	Polyclonal	Abcam	ab15580		purified	1/400 IHC
CD169	3D6.112	BioLegend	142402	AB_10916523	Purified	1/200 IHC
CD169	3D6.112	BioLegend	142408	AB_2563621	Al 647	1/200 FACS
CD169	3D6.112	BioLegend	142421	AB_2734202	BV421	1/200 FACS
CCL21	Polyclonal	R and D system	AF457		purified	1 in 50 IHC
NP-Rabbit Ig		house made				IHC
NP-PE		house made				FACS
CD16/32	93	eBioScience	14-0161-82	AB_467133	purified	1/200 FACS
CD4	RM4-5	BioLegend	100559	AB_2562608	BV510	1/300 FACS
B220	RA3-6B2	BD Biosiences	561102	AB_10561687	APC-Cy7	1/1000 FACS
B220	RA3-6B2	BD Biosiences	553092	AB_398531	APC	1/1000 FACS
B220	RA3-6B2	BD Biosiences	563793	AB_2738427	BUV395	1/800 FACS
B220	RA3-6B2	BioLegend	103240	AB_11203896	BV421	1/200 FACS
B220	RA3-6B2	BioLegend	103248	AB_2650679	BV510	1/200 FACS
CD138	281-2	BD Biosiences	558626	AB_1645216	APC	1/200 FACS
CD138	281-2	BD Biosiences	566289	AB_2739663	BV421	1/200 FACS
CD138	281-2	BioLegend	142519	AB_2562571	BV711	1/400. FACS
CD38	90	BD Biosiences	562770	AB_2737782	PerCP	1/200 FACS
CD38	90	BD Biosiences	562769	AB_2728651	Al 647	1/1000 FACS
Fas	Jo2	BD Biosiences	557653	AB_396768	PE-Cy7	1/300 FACS
Fas	Jo2	BD Biosiences	740507	AB_2740227	BV605	1/300 FACS
GL7	GL-7 (GL7)	eBioScience	48-5902-82	AB_10870775	eFluor 450	1/100 FACS
Bcl6	K112-91	BD Biosiences	563582	AB_2738292	PE-Cy7	1/300 FACS
CD62L	MEL-14	BioLegend	104441	AB_2561537	BV510	1/200 FACS
CD73	TY/11.8	eBioScience	25-0731-82	AB_10853348	PE-Cy7	1/200 FACS
CD80	16-10A1	eBioScience	15-0801-82	AB_468774	PE-Cy5	1/200 FACS
PD-L2 (CD273)	TY25	BioLegend	107203	AB_345251	Biotin	1/100 FACS
CCR7	4B12	BD Biosiences	560682	AB_1727442	PE	1/100 FACS
CD11b	M1/70	BioLegend	101263	AB_2629529	BV510	1/200 FACS
CD11c	N418	eBioScience	25-0114-82	AB_469590	PE-Cy7	1/400 FACS
CD11c	N418	eBioScience	17-0114-82	AB_469346	APC	1/500 FACS
F4/80	BM8	eBioScience	25-4801-82	AB_469653	PE-Cy7	1/400 FACS

Supplementary Table2: Secondary reagents

Target	Host		Source	Cat	Conjugated	Dilution
Rat	Donkey	polyclonal	Jackson Immunoresearch	712-545-153	Alexa Fluor 488	1/300 IHC
Rat	Donkey	polyclonal	Jackson Immunoresearch	712-165-153	Cy3	1/500 IHC
Rat	Donkey	polyclonal	Jackson Immunoresearch	712-136-153	APC	1/500 IHC
Rabbit	Donkey	polyclonal	Jackson Immunoresearch	711-095-152	FITC	1/300 IHC
Rabbit	Donkey	polyclonal	Jackson Immunoresearch	711-165-152	Cy3	1/500 IHC
Rabbit	Donkey	polyclonal	Jackson Immunoresearch	711-605-152	Alexa Fluor 647	1/500 IHC
Sheep	Donkey	polyclonal	Jackson Immunoresearch	713-545-147	Alexa Fluor 488	1/300 IHC
Goat	Donkey	polyclonal	Invitrogen	A21432	Alexa Fluor 555	1/500 IHC
Goat	Donkey	polyclonal	Jackson Immunoresearch	705-605-147	Alexa Fluor 647	1/500 IHC
RFP	Rabbit	polyclonal	Abcam	ab34771	Biotin	1/300 IHC
GFP	Rabbit	polyclonal	Invitrogen	A-21311	Alexa Fluor488	1/1000 IHC
Streptavidin alexa 555			Invitrogen	S32355		1/500 IHC
Streptavidin alexa 488			Jackson Immunoresearch	016-540-086		1/500 IHC
Streptavidin alexa 647			Jackson Immunoresearch	016-600-084		1/500 IHC
Streptavidin BV421			BioLegend	405225		1/400 FACS
Streptavidin BV711			BioLegend	405241		1/400 FACS

Supplementary Table3: Sequence of primers and probes used for qRT-PCR

Primer	Forward primer sequence (5'-3')	Reverse primer sequence (5'-3')	Probe sequence (5'-3')
Blimp-1	CAAGAATGCCAACAGGAAGTATTTT	CCATCAATGAAGTGGTGGAACTC	TCTCTGGAATAGATCCGCCA
IRF4	GGAGGACGCTGCCCTCTT	TCTGGCTTGTCGATCCCTTCT	AGGCTTGGGCATTGTTTAAAGGCAAGTTC
Aicda	GTCCGGCTAACCAGACAACTTC	GCTTTCAAAATCCCAACATACGA	GCTTTCAAAATCCCAACATACGA
Bcl6	CAGACGCACAGTGACAAACCA	ACTGCGCTCCACAAATGTTACA	ACTGCGCTCCACAAATGTTACA
S1PR1	AAATGCCCCAACGGAGACT	CTGATTTGCTGCGGCTAAATTC	
S1PR2	GGCCTAGCCAGTGCTCAGC	CCTTGGTGTAATTGTAGT	CAGAGTACCTCAATCCTGA
CCR7	GGTGGCTCTCCTTGTCATTTTC	GTGGTATTCTCGCCGATGTAGTC	TGCTTCTGCCAAGATGAGGTCACCG
CXCR5	GCTCTGCACAAGATCAATTTCTACTG	CCGTGCAGGTGATGTGGAT	CCATCGTCCATGCTGTTCACGCC
CXCR4	TGCTCCGGTAACCACCAC	CCAGAACCCACTTCTTCAGAGTAG	TAGAGCGAGTGTTGCCATGGAACC
ACKR4	TGGATCCAAGATAAAGGCGGGGTGT143YES	TGACTGGTTCAGCTCCAGAGCCATG	
β2m	CTGCAGAGTTAAGCATGCCAGTAT	ATCACATGTCTCGATCCCAGTAGA	CGAGCCCAAGACC

All primers and probes are from Eurofins Genomics (Ebersberg Germany). S1pr1 and Ackr4 were run with SYBRGreen real time PCR (Thermo Fisher Scientific). Cxcr3 (Mm00438259_m1), Ccr6 (Mm01700300_g1), Ebi2 (Mm02620906_s1) were TaqMan gene expression assays (Thermo Fisher Scientific, UK)

References:

1. Muzumdar MD, Tasic B, Miyamichi K, Li L, Luo L. A global double-fluorescent Cre reporter mouse. Genesis 45, 593-605 (2007).

2. Liberzon A, Birger C, Thorvaldsdottir H, Ghandi M, Mesirov JP, Tamayo P. The Molecular Signatures Database (MSigDB) hallmark gene set collection. Cell Syst 1, 417-425 (2015).

3. Laidlaw BJ, Schmidt TH, Green JA, Allen CD, Okada T, Cyster JG. The Eph-related tyrosine kinase ligand Ephrin-B1 marks germinal center and memory precursor B cells. J Exp Med 214, 639-649 (2017).

4. Gray EE, Friend S, Suzuki K, Phan TG, Cyster JG. Subcapsular sinus macrophage fragmentation and CD169+ bleb acquisition by closely associated IL-17-committed innate-like lymphocytes. PLoS One 7, e38258 (2012).
