# Supplementary material for: Recycling of memory B cells between germinal center and lymph node subcapsular sinus supports affinity maturation to antigenic drift
Source: Nat Commun. 2022 May 5;13:2460. doi: 10.1038/s41467-022-29978-y (PMC9072412; doi:10.1038/s41467-022-29978-y)
Supplement: Supplementary file 3 — Description of additional Supplementary File [file 41467_2022_29978_MOESM3_ESM.pdf]

Descriptions of additional supplementary Data files

Supplementary movie 1: Movement of B_{EM} between GC and SCS and inside the SCS.

Intravital microscopy. Overview of a Cγ1Cre mTmG $ACKR4^{+/+}$ drLN day 8 after foot immunization.

Cγ1Cre-dependent expression of eGFP (green) shows GC and B_{EM}. CD169 (blue) indicates location of SCS with SCS macrophages. Red: mTomato expressing stroma. Grey: second harmonic.

Supplementary movie 2: Tracking GFP^+ B_{EM} recycling between GC and SCS

Intravital microscopy of three drLN data of Cγ1Cre mTmG $ACKR4^{+/+}$ mice. eGFP-labelled B_{EM} (green), mTomato-labelled stroma (red) and CD169 labelled SCS macrophages (blue). B_{EM} were manually tracked moving from the GC towards the SCS (pink tracks) or recycling from the SCS to the GC (blue tracks).

Supplementary movie 3: Location of B_{EM} in relation to SCS macrophages and SCS lumen in wt and $ACKR4^{ko}$ drLN.

Intravital microscopy. 3D still image of SCS of a Cγ1Cre mTmG $Ackr4^{+/+}$ and $Ackr4^{-/-}$ drLN 8 d after foot immunization. Cγ1Cre-dependent expression of eGFP (green) indicating B_{EM}. CD169 (blue) SCS macrophages lining the SCS floor endothelium. Red: mTomato expressing stroma. Grey: second harmonic indicating the LN capsule. B_{EM} can be seen inside the LN parenchyma and having entered the SCS.

Supplementary movie 4: B_{EM} moving along the SCS in $ACKR4^{-/-}$ drLN

Intravital microscopy of SCS of a Cγ1Cre mTmG $ACKR4^{-/-}$ drLN 8 d after foot immunization. Cγ1Cre-dependent expression of eGFP positive B cells (green and white). CD169 (blue) SCS macrophages lining the SCS floor endothelium. Red: mTomato expressing stroma. Tracked cells (white) can be seen moving inside the SCS, but not reentering the LN parenchyma.

Supplementary movie 5: Prolonged interaction of B_{EM} with SCS macrophage

Intravital microscopy of a Cγ1Cre mTmG drLN 8 d after foot immunization. Cγ1Cre-dependent expression of eGFP (green) indicating B_{EM}. Red: mTomato expressing stroma. Blue: CD169 on SCS macrophages.

Supplementary movie 6: Colocalization of B_{EM} with increased Ca2+ and SCS macrophages

Animation merging images from Fig. 4B, showing surface rendering of $CD169^{+ve}$ macrophages (purple), FDC staining outlining a GC (orange), and $eGFP^+$ B cells (green) in one frame, and the FRET intensity of B cells in the second frame. The B cell FRET intensity is color coded from purple (low) to orange (high).

Orange B cells with Ca^{2+} FRET signal are seen in close contact with $CD169^{+ve}$ macrophages in the SCS at top edge of the lymph node section, and inside the GC.

Supplementary movie 7: B_{EM} acquiring $CD169^{+}$ material from SCS macrophage

Intravital microscopy of a Cγ1Cre mTmG drLN 8 d after foot immunization. Cγ1Cre-dependent expression of eGFP (green) indicating B_{EM}. $CD169^{+}$ material (blue) can be seen at the trailing edge of the migrating B_{EM}.

Supplementary movie 8: 360-rotation showing B_{EM} trafficking between the GC and the SCS

Light sheet microscopy shows $tdTomato^{+}$ B_{EM} from a $S1PR1^{CreERT2}$ Ai14 mouse of a, 8 d after NP-CGG foot immunization. dTomato+ BEM in red, CD169+ macrophages indicating the SCS floor in blue.

Supplementary movie 9: 360-rotation showing B_{EM} interacting with IC in SCS

Light sheet microscopy of $tdTomato^{+}$ B_{EM} (red) in a drLN of a $S1PR1^{CreERT2}$ Ai14 mouse, 10 min after injection with Alexa488 labelled immune complex (IC) into the foot. IC is shown in green. B_{EM} are seen in the GC, between the GC and interacting with IC in the SCS floor.

Supplementary movie 10: B_{EM} moving between GC and SCS

Light sheet microscopy shows $tdTomato^{+}$ B_{EM} (red) in a drLN of a $S1PR1^{CreERT2}$ Ai14 mouse, 10 min after injection with Alexa488 labelled immune complex (IC) into the foot. IC is shown in green. B_{EM} are seen travelling between the GC (left) and IC in the SCS (bottom left), and in close contact with IC in the SCS floor.
